# Harvest selection on multiple traits in the wild revealed by aquatic animal telemetry

**DOI:** 10.1002/ece3.5224

**Published:** 2019-05-20

**Authors:** Even Moland, Stephanie M. Carlson, David Villegas‐Ríos, Jørgen Ree Wiig, Esben Moland Olsen

**Affiliations:** ^1^ Flødevigen Institute of Marine Research His Norway; ^2^ Department of Natural Sciences, Centre for Coastal Research University of Agder Kristiansand Norway; ^3^ Department of Environmental Science, Policy and Management University of California Berkeley California; ^4^ Department of Ecology and Marine Resources, Ichthyology Group IMEDEA, Instituto Mediterráneo de Estudios Avanzados Esporles Spain; ^5^ Department of Ecology and Marine Resources, Fisheries Ecology Group Instituto de Investigaciones Marinas (IIM‐CSIC) Vigo, Pontevedra Spain; ^6^ Directorate of Fisheries Bergen Norway

**Keywords:** acoustic telemetry, catchability, Decapoda, European lobster, fishery selection, home range, movement, repeatability, vulnerability

## Abstract

Harvesting can have profound impacts on the ecology and evolution of marine populations. However, little is known about the strength and direction of fisheries‐induced selection acting on multiple traits in the wild. Here, we used acoustic telemetry to directly monitor individual behavior and fate in an intensively harvested species, the European lobster (*Homarus gammarus*, *n* = 100), in southern Norway. Overall, 24% of the tracked lobsters survived the two‐month harvest season within the study area. Our results indicated that local survival was not random with respect to phenotype. We found no clear support for fisheries‐induced selection acting directly on body size. However, lobsters with large crusher claws relative to their body size, typical of socially dominant individuals, appeared at higher risk of being captured in the conventional trap fishery. We also detected a fine‐scale spatial gradient in survival. After accounting for this gradient, individuals displaying larger home ranges were more likely to survive the harvest season. Finally, we found significant repeatabilities for lobster behavior on a monthly timescale, indicating that individual behavioral attributes tended to persist and may reflect personality. Our study therefore provides empirical support for the need to consider an evolutionary enlightened approach to fisheries management that considers the influence of harvest on multiple traits of target species.

## 1 INTRODUCTION

Human harvesting of marine wildlife dates back millennia and has had significant impacts on populations and ecosystems (Jackson et al., 2001; Limburg, Walther, Hong, Olson, & Storå, 2008; Myers & Worm, 2003). Often, harvesting tends to drive selection on body size because of preference, gear selectivity, or regulations imposing size limits. Similarly, morphological traits independent of body size per se may also be under selection in different fishing gear, by, for example, affecting the probability of becoming trapped in a net or biting a hook (Alós, Palmer, Linde‐Medina, & Arlinghaus, 2014; Kuparinen, Kuikka, & Merilä, 2009; Reis & Pawson, 1999). A more unappreciated ecological impact on the targeted populations is the removal of individuals displaying certain behaviors that make them more vulnerable to the fishery (Uusi‐Heikkilä, Wolter, Klefoth, & Arlinghaus, 2008; but see: Klefoth, Skov, Kuparinen, & Arlinghaus, 2017). For instance, highly active individuals may encounter static fishing gear more frequently and so exhibit greater catchability (Alós, Palmer, & Arlinghaus, 2012; Rudstam, Magnuson, & Tonn, 1984). Intriguingly, selection on life‐history, behavioral, morphological, and physiological traits may occur simultaneously, and understanding the ultimate outcome of correlational or indirect selection on multiple traits hinges on good information about individual performance, and how this scale to spatial and temporal variation in fisher behavior and the resulting harvest pressure (Alós et al., 2012; Hollins et al., 2018; Olsen, Heupel, Simpfendorfer, & Moland, 2012; Villegas‐Ríos et al., 2014). From an evolutionary perspective, selective harvesting may lead to contemporary evolutionary changes in the harvested populations if the selected traits are heritable (Biro & Post, 2008; Heino; , Pauli, & Dieckmann, 2015; Swain, Sinclair, & Hanson, 2007). Although such genetic changes may, under certain circumstances, be reversed (Conover, Much, & Arnott, 2009; Reznick, Bryga, & Endler, 1990), maladaptive changes in multiple traits due to fishing can leave populations at a “point of no return” impeding their recovery (Walsh, Munch, Chiba, & Conover, 2006) and may also cause long‐lasting negative effects on the resilience of harvested ecosystems to fishing and environmental change (Kuparinen, Boit, Valdovinos, Lassaux, & Martinez, 2016).

Understanding how selection and evolution of multiple traits operate in nature is logistically challenging, especially in the aquatic realm, where animals cannot be observed easily. Whereas some life‐history and morphological traits can be obtained readily from captured individuals, assessment of behavior and survival in the wild requires tracking of individual movements, and fate, over large spatial and temporal scales. Here, we used acoustic telemetry to track a subset of a European lobster (*Hommarus gammarus*) population and investigated harvest selection on multiple traits under high recreational and commercial fishing pressure (Kleiven, Olsen, & Vølstad, 2012). Lobster populations in southern Norway are especially well suited for exploring the influence of harvest on behavioral phenotypes because lobsters move relatively little (Moland, Olsen, Knutsen, et al., 2011) and the fate of individual lobsters can be determined from telemetry records (Olsen et al., 2012). As a basis for our study, Wiig, Moland, Haugen, and Olsen (2013) demonstrated that the fate of lobsters during the fishing season depended on the location of the home range relative to areas subject to high fishing pressure. In general, when fishers are not fixed in their behavior and change the location of the traps, as is the case in the southern Norway lobster fishery, individuals with higher activity are more prone to being caught (Alós et al., 2012). Building on the earlier work in this system, we developed a series of hypotheses to explore the influence of harvest on multiple traits. Our *first hypothesis* is that lobsters that displayed larger vertical and/or horizontal movements would be more likely to be captured in the fishery; however, we expected no clear effect of home‐range size per se on survival in this fishery because fishers change the location of their traps. Other traits, like body size or claw size, are known to determine social status, dominance, and competitive ability in lobsters (Atema & Cobb, 1980; Elner & Campbell, 1981; Skog, 2009; Sørdalen et al., 2018) and crustaceans in general (Bywater, Angilletta, & Wilson, 2008) and can therefore affect fitness as well (Lee & Seed, 1992; Scrivener, 1971). However, long‐term data from field studies exploring the relationship between individual traits and vulnerability to capture are scarce. Our *second hypothesis* is that lobsters with larger claws, potentially reflecting socially dominant individuals, will be more prone to protecting a baited trap, which should increase their vulnerability to capture. However, we hypothesized (*third hypothesis*) that larger lobsters may experience reduced harvest selection because of the diameter of the rigid trap entrance used in the lobster fishery, which may physically limit the ability of very large lobsters to enter the trap and be captured. While life‐history and morphological traits often have moderate heritabilities (Mosseau and Roff, 1987), the heritability of spatial behavior of wild animals has never been assessed. In this situation, a valid substitute of heritability is the repeatability of behavior (Dochtermann, Schwab, & Sih, 2015). Some pioneering studies on repeatability of behavior demonstrated that spatial ecology traits such as home range are repeatable in aquatic species like burbot (*Lota lota*; Harrison et al., 2015) and cod (*Gadus morhua*, Villegas‐Ríos, Reale, Freitas, Moland, & Olsen, 2017; Villegas‐Ríos, Réale, Freitas, Moland, & Olsen, 2018), and other studies revealed the repeatability of behavior in decapods based on laboratory assays (e.g., Gherardi, Aquiloni, & Tricarico, 2012). With this background, our *last hypothesis* is that spatial behavioral traits of lobster are repeatable, and thus, selection on behavioral traits has the potential to fuel evolutionary changes.

## 2 MATERIALS AND METHODS

### 2.1 Study species

The European lobster is a large long‐lived decapod crustacean of ecological and commercial importance, distributed from northern Norway to Morocco in North Africa (Triantafyllidis et al., 2005). The species is considered a nocturnal animal, where light hours are generally spent solitary inside shelters on rocky bottoms (Mehrtens, Stolpmann, Buchholz, Hagen, & Saborowski, 2005; Moland, Olsen, Knutsen, et al., 2011; Smith, Collins, & Jensen, 1998, 1999). European lobsters rarely move more than a few kilometers for periods up to multiple years (Agnalt, Kristiansen, & Jørstad, 2007; Dannevig, 1936; Huserbråten et al., 2013; Smith, Jensen, Collins, & Mattey, 2001). Longevity potentially spans several decades (Sheehy, Bannister, Wickins, & Shelton, 1999). In Norway, fishery catch per unit effort has decreased by 65% from the 1950s to 2000s (Kleiven et al., 2012). Since 2008, lobsters in Norway are legally caught in traps fitted with two circular escape vents measuring 60 mm in diameter during a two‐month season (1 October–30 November). The trap type mostly employed by fishers is the two‐chambered “parlor trap,” with two round entrance funnels mounted on the traps’ vertical side walls. The standard dimensions of the inner section of an entrance funnel are 120 mm diameter. Fishers typically move and rebait traps every 1–3 days in the beginning of the fishery, but hauling occurs less frequently toward to end of the season. Although fishers move traps, areas regarded as good lobster habitat are fished more intensively (Wiig et al., 2013). Minimum legal size is 25 cm total length (TL, measured from the tip of the rostrum to the end of the middle uropod, ≈90 mm carapace length, CL), and there is a trade‐and‐landings ban on egg‐bearing females. Effort (total number of traps deployed) is limited to 10 and 100 traps per person for recreational and commercial participants, respectively. As of 2017, a slot‐limit harvesting rule with a maximum total length of 32 cm was introduced in the fishery to protect size and age structure.

### 2.2 Study system

**Figure 1:**
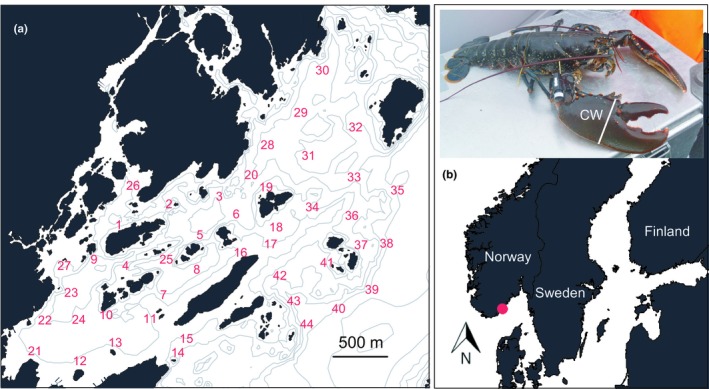
Study area: the Sømskilen basin and nearby islands (a) on the Norwegian Skagerrak coast (b). Insert in panel (b): a male European lobster equipped with a Vemco V13P‐L acoustic transmitter. CW: claw width. Isobaths shown are the 5‐, 10‐, 20‐, 30‐, 50‐, 100‐, and 150‐m depth contours. Numbers in (a) denote GPS positions of 44 Vemco VR2W acoustic receivers deployed to receive signals sent by acoustic transmitters

This study was conducted within a coastal archipelago on the Norwegian Skagerrak coast (58°24′N 8°45′E) (Figure 1). Maximum depth is 50 m, and habitats are diverse, including exposed and submerged islands, boulder fields, flats consisting of soft sediment, eel grass beds, and kelp forest (Olsen & Moland, 2011). A partly submerged glacial moraine cuts through the area parallel to the coastline, forming a rock reef consisting of variable‐sized cobble. The habitat found in the area is representative of that found along most of the Norwegian Skagerrak coast, of which large swathes may be considered good lobster habitat (Moland, Olsen, Andvord, Stenseth, & Knutsen, 2011; Moland, Olsen, Knutsen, et al., 2011). Due to its proximity to human population centers and its multitude of sheltered locations, this part of the coastline is popular for both commercial and recreational lobster fishers.

### 2.3 Tagging and monitoring

A total of 100 male wild lobsters were captured in the study area from 1 to 31 August in 2011 (*n* = 50) and 2012 (*n* = 50). Only males were selected to ensure that tagged individuals recovered by fishers would be kept (and subsequently reported). Individuals were collected for tagging by using two types of “parlor” lobster traps that were baited with frozen Atlantic mackerel (*Scomber scombrus*): standard traps (entrance diameter 120 mm) and “extra‐large” traps (entrance diameter 180 mm). Soak time varied from 1 to 4 days. For all individuals, we recorded capture location using global positioning system (GPS) and measured carapace length (CL, mm); total length (TL, mm); length, width, and height of crusher claw propodite (mm); and width of abdomen (mm) using vernier calipers (Table 1). For acoustic monitoring of lobster behavior and fate, lobsters were equipped with acoustic transmitters (Vemco V13P–L, diameter 13 mm length 36 mm, weight in seawater < 6 g, Vemco Divison, Amirix Systems Inc., Halifax, Canada). Tags were programmed to transmit signals (69 kHz) at 110–250 s random intervals (mean 180 s), coded with an ID number to distinguish among individuals. Also, the transmitters were equipped with a pressure‐sensitive transducer (0.22 m resolution, 2.5 m accuracy and 50 m maximum depth) to obtain information about lobster depth use. Transmitters were attached to lobsters by means of a harness made of a cable tie and soft plastic tubing in which the transmitter and a T‐bar tag were inserted (TBA1, 45 mm × 2 mm, Hallprint Pty. Ltd, Holden Hill, South Australia). This harness was attached between the most robust denticles on the middle segment (*carpus*) of the crusher claw limb (Moland, Olsen, Andvord, et al., 2011; Wiig et al., 2013). To maximize the return rate of recovered tags from fishers, the T‐bar tag informed fishers that a 5€ reward would be paid if returned to the Institute of Marine Research. As a consequence, it was possible to confirm whether individuals were captured or not as a supplement to the collected acoustic data. After tagging, lobsters were released from the surface at their GPS capture location. The total handling time was 5–15 min, depending on the number of lobsters caught in each trap. A total of 44 acoustic receivers (VR2W, Vemco Divison, Amirix Systems Inc., Halifax, Canada) were moored throughout the study area (geographic coverage ≈ 3 km^2^) and positioned at 3 m depth using subsurface trawl floats (Figure 1). Data stored within receivers were downloaded early in December in both years, after the end of the lobster fishing season (30 November). Transmitters were lost if lobsters molted during the course of the study (see below).

**Table 1:** Summary statistics of 72 male European lobster (*Homarus gammarus)* equipped with acoustic transmitters in August 2011 and 2012 and included in the present analyses

Traits	Range	Mean	*SD*
TL (cm)	25.0–39.5	27.6	23.3
CL (mm)	88–152	99.1	9.8
Claw W (mm)	36–104	52.8	8.7
HR (m^2^)	22,733–638,216	173,053	125,887
Dist (m)	6,513–175,806	75,200	38,169
Depth (m)	5–38.2	17.9	8.3
Amp (m)	1.1–24.3	10.1	4.7

Note

Minimum legal size in Norway is 25 cm TL.

Abbreviations: Morphological traits: Amp, depth amplitude; CL, carapace length; Claw W, crusher claw width; TL, total length. Behavioral traits on a monthly basis as estimated for the month of September: Depth, mean depth; Dist, cumulative distance; HR, home range (UD95).

**Figure 2:**
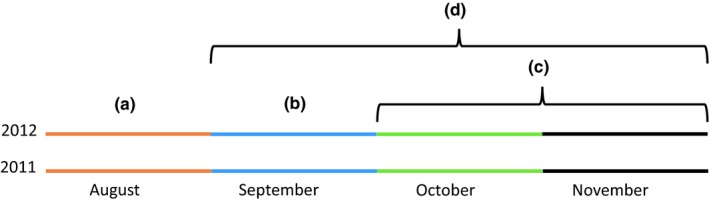
Time‐line visualization of the study period during autumn 2011 and 2012. August (a): trap capture and acoustic tagging of male European lobster. September (b): one‐month common observation period for all individuals tagged and present, undisturbed by trapping. Behavioral traits quantified for September (b) were used in selection analyses. October–November (c): period of ordinary lobster fishing and selection, traps mapped and counted in 2011. September–October–November (d): monthly behavioral traits quantified and used in repeatability estimates

During the 2011 lobster fishing season, we used a handheld GPS to record the positions of all observed lobster trap surface buoys in the study area, including those deployed by recreational and commercial fishers (see Wiig et al., 2013). Trap counting continued throughout the fishing season three times per week in October and two times per week in November 2011. The last day of trap counting was 28 November 2011. For a time‐line visualization of the study, see Figure 2.

### 2.4 Data analyses

**Figure 3:**
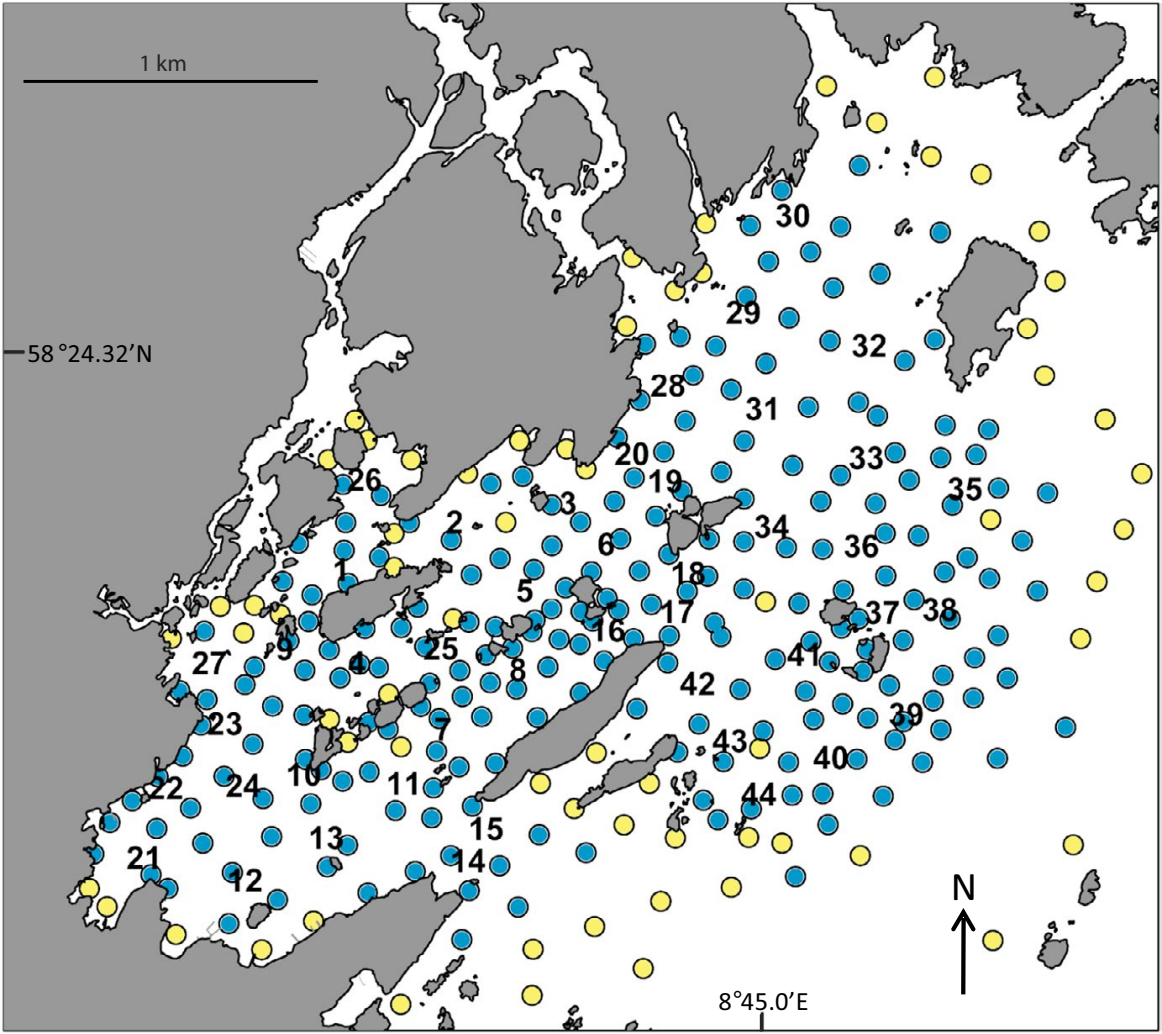
Acoustic range testing in the 5 km^2^ study area used for monitoring movement and fates of European lobster (see also Figure 1), showing positions where a range test tag was deployed and detected (blue circles) or not detected (yellow circles) by one or more of the acoustic receivers (numbers 1–44)

The presence and movement of lobsters within the receiver array were determined from detections at multiple receivers and depths through time. Overlap in receiver detection range was evaluated by a range test conducted prior to the study. The configuration and detection range of receivers prevented disappearance of individuals within the array (Wiig et al., 2013). As a consequence, all tagged lobsters that were alive and moving within the study area were highly likely to be detected by the receiver array (Figure 3). Based on horizontal and vertical movement data, and information from fishers, the following mutually exclusive fates were determined for all lobsters at the end of the 2011 and 2012 fishing season: (a) harvested, (b) molted, (c) dispersed out of study area, and (d) survived within study area (Wiig et al., 2013).

There are no likely natural predators on adult lobsters in the area, and mortality due to disease or senescence was deemed unlikely given study duration (maximum of four months). However, we note that molting could be confounded with natural mortality. A lobster was classified as harvested when the signal disappeared from the study area and confirmed by a fisher returning the tag or if the individual showed the typical signs of being locked in a trap (i.e., fixed depth and position) prior to signal disappearance, without subsequent tag return from fishers. A lobster was classified as molted when the data showed permanent cessation of movement at constant depth for at least seven days, lasting to the end of the study. A lobster was classified as dispersed when the signal disappeared from one of the outermost receivers. Lastly, a lobster was classified as surviving when horizontal and vertical movement continued throughout the fishing season. While environmental factors such as short‐term variability in water temperature, wind conditions, and lunar periodicity have been shown to influence catchability of lobsters (see, e.g., Drinkwater, Tremblay, & Comeau, 2006), these factors would be shared among all individuals within a given year and so are not the focus of our work. Our principal aim was to quantify individual differences in survival (i.e., selection), not changes in average capture rates across different environmental conditions.

To quantify lobster behavior, we first used the algorithm developed by Simpfendorfer, Heupel, and Hueter (2002) to estimate the mean horizontal position (latitude and longitude) of each lobster during consecutive 30‐min time intervals based on the logged receiver data. The method does not provide an exact location of an animal at a given time, but rather estimates short‐term (30 min) centers of activity. The vertical location was determined as mean depth for the same 30‐min time intervals. We then used these data to characterize movement behavior and home ranges at a monthly scale. Monthly values of the different behavioral metrics were only calculated when the lobster was present in the array for at least 20 days, not necessarily consecutive, in a particular month.

We estimated two measures of vertical activity. First, monthly depth position was estimated as the average depth in a particular month. Second, monthly depth amplitude was estimated as the average daily depth amplitude (daily observed maximum depth minus daily observed minimum depth; Freitas, Olsen, Moland, Ciannelli, & Knutsen, 2015). Then, monthly cumulative horizontal movement was calculated as a proxy of horizontal movements by adding distances among all centers of activity. Plotting cumulative horizontal movement against number of 30‐min centers of activity revealed a positive distance bias (i.e., lobsters with more detections appeared to move farther). As detection rates were lowered when lobsters moved in rocky and rugose habitats, some corridors of movement would yield less detections and consequently fewer 30‐min centers of activity. For the purpose of unbiased comparison between individuals moving in heterogenous habitat, we divided the monthly cumulative horizontal movement distance by the number of 30‐min centers of activity calculated for each individual in each month. Last, monthly home ranges were quantified as a measure of space use as the smallest area containing 95% of the utilization distribution (UD95) of an individual (Rogers & White, 2001). The same smoothing factor (*h*0 = 50) was used to estimate the home range of all animals.

### 2.5 Repeatability of behavioral traits

Monthly metrics quantified for individuals surviving through the two‐month harvest season (i.e., October and November, see Figure 2) were used to test for repeatability (consistency in individual behavioral traits). Bayesian univariate mixed‐effects models were fitted with the following response variables: *home range*, *cumulative horizontal movement*, *vertical position*, and *depth amplitude*. These four traits were chosen as they covered the range of metrics quantified. When appropriate, response variables were log‐transformed. Individual identity was used as a random effect. To obtain adjusted (unbiased) estimates of repeatability, the following covariates were tested as confounding variables: claw width (continuous; centered and scaled), month (categorical; three levels: September, October, and November), year (categorical; two levels: 2011 and 2012), and all two‐way interactions. We further included the mean latitude and longitude for each month as fixed effects to avoid pseudo‐repeatability due to differences in habitat properties derived from the location where each lobster was tagged. For each model, we used weakly informative inverse‐gamma distribution priors (Hadfield, 2010) although changing the priors had little to no effect on the repeatability estimates (data not shown). We ran a total 500,000 iterations with a 10,000 burn‐in and thinning every 100 iterations for each model. We checked for proper model mixing and convergence by inspecting the autocorrelation and posterior distributions of the model effects. Support for the significance of the fixed and random effects was based upon comparisons of DIC values between models where the effect of interest was included versus excluded (Hadfield, 2010). Repeatability (*r*) estimates and associated 95% credible intervals (CI) for each response variable were calculated based on the posterior distributions from the most parsimonious Bayesian mixed models described above (Dingemanse & Dochtermann, 2013), using the formula:

$$r=V_{\text{ind}}/\left(V_{\text{ind}}+V_{\text{res}}\right)$$

where V_ind_ is the variance across random intercepts and V_res_ is the residual variance. Since in all cases the most parsimonious model included some significant fixed effects, the repeatability values we provide correspond to adjusted repeatabilities (Nakagawa & Schielzeth, 2010). Adjusted repeatabilities with CIs nonoverlapping 0 were considered significant, and therefore, the trait deemed repeatable (Dingemanse & Dochtermann, 2013; Nakagawa & Schielzeth, 2010). Bayesian mixed‐modeling and repeatability estimation were conducted in the statistical software R (R Development Core Team, 2016) using the library MCMCglmm (Hadfield, 2010).

### 2.6 Harvest selection

For the selection analyses, we included the behavioral traits quantified for the month of September (the month prior to the lobster fishing season, see Figure 2), during which all lobsters were alive, and their behavior would be unaffected by the presence of baited traps. In addition, we included the two morphological traits *body size* (TL) and *claw width*. Claw width was strongly correlated with body size (*r* = 0.78). Therefore, we first calculated the residuals from a linear regression of claw width on body length and then used these residuals in further analyses as an estimate of relative claw size after controlling for body size.

When modeling survival (i.e., fitness), we started with the Lande–Arnold linear regression approach (Lande & Arnold, 1983) using relative longevity (days survived/mean days survived, *D*_surv_) as response variable because there were so few survivors (*N* = 17). We note that lobsters were probably able to mate (increase fitness) during the fishing season (mating season extends from month 1 to month 2), so extended survival might transfer into extended mating opportunities. Choices of explanatory variables were based on the working hypotheses linked to life history, morphology, and behavior. To facilitate comparison with other studies, all predictor variables were standardized to a mean of zero and a standard deviation of one (to obtain the variance‐standardized selection gradients). Our starting model (prior to model selection) had the following structure:

$$D_{\text{surv}}=\beta_{0}+\beta_{1}\text{TL}+\beta_{2}\text{RCW}+\beta_{3}\text{HR}+\beta_{4}\text{DA}+\beta_{5}\text{LON}+\beta_{6,\text{Y}}$$

and included slope parameters (*β*) for total length (TL), relative claw width (RCW), home‐range size (HR), depth amplitude (DA), mean longitude (LON), and year (Y) added as a factor as sampling included data collected in both 2011 and 2012. Monthly cumulative horizontal movement and mean latitude were strongly correlated with home range (Table 2) and thus not included as explanatory variables. Similarly, mean depth was excluded because it was strongly correlated with depth amplitude (Table 2). We used Akaike's information criterion (AIC) to select the model structure that best‐balanced bias and variance (Burnham & Anderson, 1998). For comparison, we also estimated the mean‐standardized selection gradients as defined by Hereford, Hansen, and Houle (2004) and recommended by Matsumura, Arlinghaus, and Dieckmann (2012). These regressions with multiple explanatory variables (traits) estimate the strength of selection acting directly on each trait, independent of any correlation with other traits included in the model (Brodie, Moore, & Janzen, 1995). In addition, we used linear regressions to describe the total strength of selection acting on each trait, including any indirect selection acting through correlated traits.

**Table 2:** Correlations among standardized lobster traits on a monthly basis as estimated for September

Traits	TL	RClaw	HR	Dist	Depth	Amp	Lon	Lat
TL	1	0.00	0.26	0.1	0.34	0.29	0.05	0.31
RClaw	0.00	1	0.09	0.10	−0.21	−0.06	0.11	0.04
HR	0.26	0.09	1	0.64	0.21	0.16	0.09	0.51
Dist	0.1	0.10	0.64	1	0.24	0.12	0.17	0.42
Depth	0.34	−0.21	0.21	0.24	1	0.65	0.39	0.01
Amp	0.29	−0.06	0.16	0.12	0.65	1	0.28	−0.03
Lon	0.05	0.11	0.09	0.17	0.39	0.28	1	−0.02
Lat	0.31	0.04	0.51	0.42	0.01	−0.03	−0.02	1

Abbreviations: Amp: depth amplitude; Depth: mean depth; Dist: cumulative distance; HR: home range; Lat: mean latitude; Lon: mean longitude; RClaw: relative crusher claw width; TL: total length.

Lastly, we applied the Janzen–Stern logistic regression approach (Janzen & Stern, 1998) for estimating approximate selection gradients (*β*_avggrad_), using the same set of explanatory variables as in the most parsimonious linear regression model. Here, survival (*S*) was used as response variable, corresponding to an absolute fitness of one (survived) or zero (harvested), again using standardized trait values. The analyses were performed using the *glm* function in the statistical software R (R Development Core Team, 2016).

## 3 RESULTS

Eight and nine individuals were censored from analyses due to molting or tag malfunction in 2011 and 2012, respectively. In addition, four and seven individuals traversed outside the detection range of receivers in 2011 and 2012, respectively, preventing accurate estimates of home‐range size and movement metrics, so these individuals were excluded. After these exclusions, 72 lobsters in total were included in our analyses (Table 1). Out of these, seven and 10 were active (i.e., had survived) after the fishing season had ended in 2011 and 2012, respectively. The remainder (*N* = 55) were classified as harvested based on tag return from fishers (*N* = 49) or on visual examination of depth data recorded prior to signal cessation (*N* = 6). When including all tag returns from fishers (including those from lobsters that had traversed outside of the study area), fishing mortality in the tagged population amounted to 83% in 2011 (Wiig et al., 2013) and 76% in 2012 (this study).

**Figure 4:**
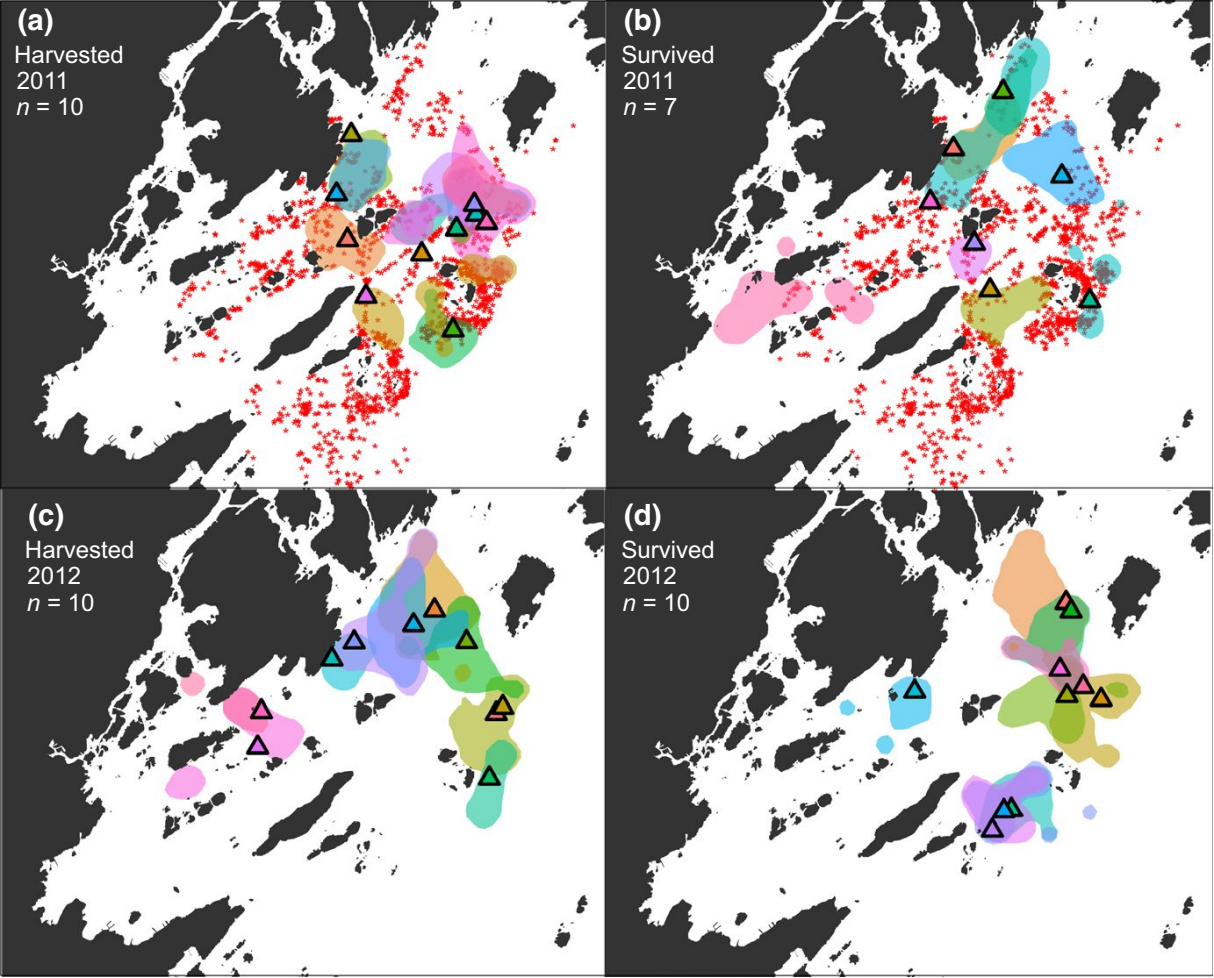
September UD95 home ranges for European lobster harvested (a and c) and surviving (b and d) the 2011 (a–b) and 2012 (c–d) fishing seasons (1 October–30 November) estimated from acoustic monitoring. For illustration purposes, a randomly selected subset of 10 home‐range estimates from the harvested individuals included in the analyses (*n* = 55) are shown in each of panel (a) and (c), while home ranges for all survivors (*n* = 17) are included in (b) and (d). Pyramids are capture and release locations of individuals shown in corresponding color. Red asterisks (a–b) mark the cumulative GPS locations of all lobster traps deployed in the study area during the 2011 fishing season (see Wiig et al., 2013). For bathymetric information, see Figure 1

A cumulative total of 4,781 lobster trap sets were registered in the study area throughout the 2011 fishing season, with a mean of 78 traps per day. Overall fishing activity peaked early in the season (Wiig et al., 2013). There was also a marked fine‐scale spatial variation in fishing activity, where the highest density of traps was found around islands in the outer, eastern, part of the study area (Figure 4).

In total, the 72 lobsters included in our analyses yielded 592,766 detections during their common observation period of 30 days in the month of September. Individual detections ranged from 788 to 19,360 (mean 8,233 ± 553 SE), constituting 0.13–3.27% of all detections.

**Figure 5:**
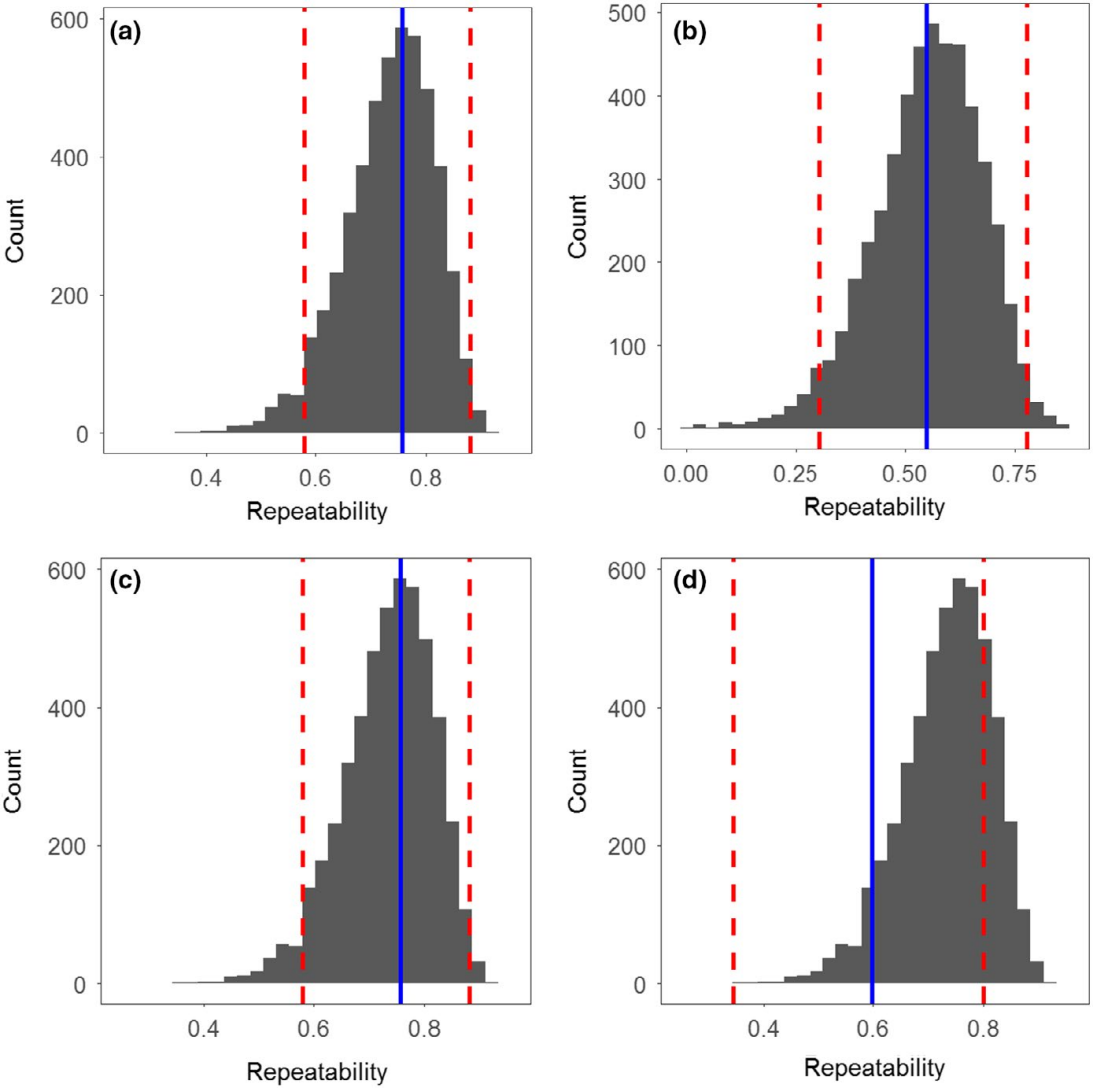
Posterior distributions of the adjusted repeatability of (a) home range, (b) cumulative distance, (c) mean depth, and (d) depth amplitude as estimated from Bayesian mixed‐effects models, showing the posterior mean (blue line) and the 95% confidence interval (red dashed lines). See also Table S1

Home‐range sizes ranged from 22,733 to 638,216 m^2^ in September (mean 173,053 m^2^ ± 20,395 SE). For a qualitative comparison of the home‐range locations and sizes, we plotted the estimated home ranges of all lobsters that had survived the harvest season in 2011 (*N* = 7; Figure 4b) and 2012 (*N* = 10; Figure 4d), as well as the estimated home ranges of a subset of 10 lobsters selected at random from lobsters that were harvested in 2011 (Figure 4a) and 2012 (Figure 4c). The mean depth occupied by lobsters during the same period ranged from 5 to 38.2 m (mean 17.9 m ± 2.1 SE). The main movement metrics *cumulative horizontal movement* (DI), *home‐range size* (HR), *vertical position* (DM, daily mean depth), and *depth amplitude* (DA, daily maximum depth − daily minimum depth) quantified for individuals surviving through October and November revealed significant adjusted repeatabilities (range 0.55–0.76; Table 3 and Figure 5), indicating that individual lobsters displayed consistent behaviors through time (Table S1).

**Table 3:** Adjusted repeatability of lobster behavioral traits (DA, depth amplitude; DI, cumulative distance; DM, mean depth; HR, home range) estimated from univariate Bayesian mixed‐effects models

Traits	Lower	adj‐*r*	Upper	Significant effects	Nonsignificant effects
HR	0.58	**0.76**	0.89	Month	Year, Claw width, Year:Month, Claw width:Month, Year:Claw width, latitude, longitude
DI	0.30	**0.55**	0.78	Month, Claw width	Year, Year:Month, Claw width:Month, Year:Claw width, latitude, longitude
DM	0.58	**0.76**	0.88	Month, longitude	Year, Year:Month, Claw width:Month, Year:Claw width, latitude
DA	0.34	**0.60**	0.80	Month, Claw width, Year:Claw width	Year, Claw width, Year:Month, Claw width:Month, latitude, longitude

Note

Significant adjusted repeatabilities (adj‐*r*) are shown in bold, with associated 95% CI. A total of 70 replicates from 28 individuals were used, with a mean number of 2.5 replicates per individual.

**Figure 6:**
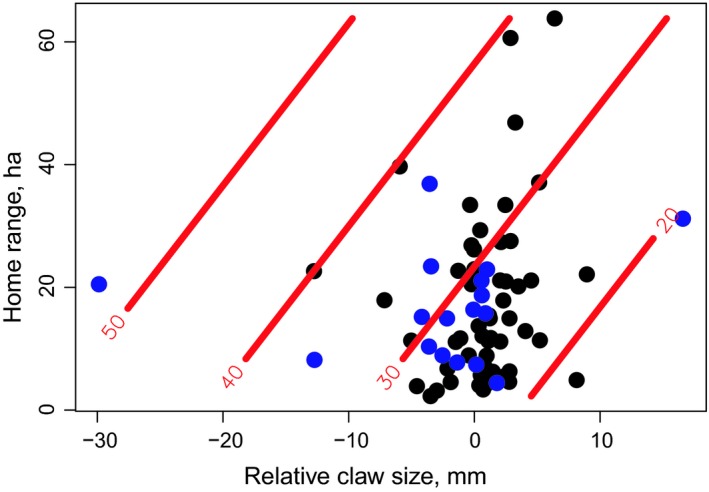
Lobster survival during the harvest season (days) as predicted from the most parsimonious linear model including relative claw width and home‐range size (UD95) as explanatory variables (see Results). Predictions (isolines) are for the mean observed longitudinal position, showing how lobster survival tended to be higher for individuals with small crusher claws (relative to their body length) and larger home ranges. For ease of interpretation, the model producing this plot was based on observed, untransformed values of survival, behavior, and morphology (blue circles: uncaught lobsters, black circles: lobsters caught in the fishery). Home ranges are displayed in hectares (ha)

The most parsimonious model describing the strength of fishery‐induced selection acting directly on each lobster trait supported additive effects of relative claw width and home‐range size on the days survived during the harvest season (Table 4). Accounting for a spatial gradient where lobsters in the western part of the study area tended to survive at a higher rate (*β*_Longitude_ = −0.21, SE = 0.09, *p* = 0.02), harvesting apparently selected against male lobsters with large crusher claw size (for their body size) and individuals with small home range (Table 4, Figure 6). Removing the effect of crusher claw size increased the AIC by only 1.1 units (Table 4), indicating that both models had some support (differing by <2 units). However, to provide estimates of selection, we relied on the model with the lowest AIC score (Burnham & Anderson, 1998). Removing the effect of home‐range size increased the AIC by 3.5 units, so the support for this simplified model was weak (Table 4). Note also that the best model outperformed the null model (no effects) by more than 7 AIC units (Table 4). Linear regressions confirmed that the total strength and direction of fisheries‐induced selection seemed to favor individuals with larger home ranges (*β*_Home range_ = 0.18, SE = 0.09, *p* = 0.06) and relatively small crusher claws (*β*_Claw width_ = −0.16, SE = 0.09, *p* = 0.09). The strength and direction of the total fisheries‐induced selection acting on body size and vertical movement was less clear (*β*_Total length_ = 0.15, SE = 0.09, *p* = 0.11; *β*_Depth amplitude_ = −0.08, SE = 0.09, *p* = 0.38). When applying the Janzen–Stern logistic regression approach, harvesting selected against male lobsters with large crusher claw size, while favoring individuals with large home‐range size (Table 5).

**Table 4:** Lande–Arnold linear regression modeling of lobster survival during the 2011 and 2012 fishing season (1 October–30 November) using relative longevity as the response variable (see Materials and Methods)

Model no.	Model structure	Dev	P	AIC
1	TL + RCW + HR + DA + LON + Y	34.79	7	167.95
2	TL + RCW + HR + DA + LON	35.40	6	167.20
3	TL + RCW + HR + LON	36.13	5	166.68
**4**	**RCW + HR + LON**	**36.98**	**4**	**166.35**
5	HR + LON	38.63	3	167.49
6	RCW + LON	40.03	3	170.05
7	NULL	44.45	1	173.6

Note

The model with lowest AIC is indicated in bold.

Abbreviations: AIC, Akaike's information criterion score; Dev, deviance; P, number of parameters. Explanatory variables (standardized): CW, claw width; DA, depth amplitude; DD, daytime depth amplitude; DI, cumulative distance (adjusted, see Materials and Methods); DM, mean depth; HR, home range (UD95); TL, total length; Y, year added as a factor since sampling included both 2011 and 2012.

**Table 5:** Harvest selection acting on European lobster behavior and morphology during the 2011 and 2012 fishing season, estimated from the Lande–Arnold linear regression approach (a) and the Janzen–Stern logistic regression approach (b). Explanatory variables are from the most parsimonious linear regression model (see Table 4). Linear regression: *SD*‐standardized selection gradients (*β_SD_*) with standard errors (SE) and *p*‐values and also the mean‐standardized selection gradient (*β_μ_*) for home range. Logistic regression: regression coefficients (*α*) with standard errors (SE) and p‐values and also the approximate selection gradients (*β*_avggrad_)

(a) Lande–Arnold linear regression approach				
Variables	*β_SD_*	SE	*p*	*β_μ_*
Relative claw width (RCW)	−0.15	0.09	0.09	–
Home range (HR)	0.21	0.09	0.02	3.27

(b) Janzen–Stern logistic regression approach				
Variables	Α	SE	*p*	*β*_avggrad_
Relative claw width (RCW)	–0.55	0.31	0.08	−0.37
Home range (HR)	0.23	0.31	0.45	0.16

## 4 DISCUSSION

Our study provides strong empirical support for the expectation that passive gear fisheries may impose selection on morphology and behavior of target species (Alós et al., 2012; Heino et al., 2015; Pauli, Wiech, Heino, & Utne‐Palm, 2015). Harvesting indirectly selected against lobsters with large crusher claw size (relative to body size), which represents an indicator of social dominance (Skog, 2009). Contrary to our a priori hypothesis, harvesting also selected against small home ranges, after accounting for a spatial gradient in survival. We note that our inferences are drawn from a catchable subset of the population, as all tagged lobsters were caught in our traps prior to the onset of the regular harvest season. Therefore, the results of the study should be interpreted in terms of lobsters that are already vulnerable to being captured.

Our best‐supported model suggested that lobsters with a large crusher claw relative to body size were selected against in the trap fishery. Besides being used as tools in foraging and excavating, claws (chelae) are used in fighting and threat displays (Atema & Cobb, 1980; Elner & Campbell, 1981; Skog, 2009). To the extent that claw size is an indication of social dominance, it is possible that this result is an indirect effect of dominant and aggressive individuals being more prone to chasing off competing lobsters from around baited traps, or from within the traps once caught (protecting bait)—both of which should increase the vulnerability of dominant individuals to the trap fishery. This possibility is supported by the findings of Addison (1995) who showed that if one lobster was already caught in a trap, it was less likely that another would enter. The same study also noted that interaction between conspecifics and other species outside traps has a major impact on an individual's catchability (see also Cobb & Wang, 2012). However, our finding that harvesting selected against large relative claw size is perhaps most important in relation to sexual selection. In a recent study, Sørdalen et al. (2018) used genetic parentage assignment to compare mating success in male European lobster in protected and harvested areas. Their work clearly demonstrated a positive size‐assortative mating pattern, where larger females had tended to mate with comparatively larger males. Moreover, the study found that sexual selection acted positively on both body size and claw size in the protected population, with selection acting stronger on relative claw size. In the present study, we found harvest selection to act in the exact opposite direction: favoring small claw size relative to body size. Thus, harvesting seems to remove the larger male individuals, and importantly, the associated traits that are favored by sexual selection.

Home‐range size also influenced the probability of surviving the fishery, although in a way somewhat contrary to our a priori hypotheses that selection for this behavioral trait would be weak or absent, building on the theoretical predictions by Alós et al. (2012). In our best‐supported model (Table 4), home range was the only movement or activity‐related predictor variable included, with selection favoring individuals with large home ranges. This result is interesting and should be interpreted in light of the observation that all our study animals should be considered trappable based on the fact that all were caught at least once, during our initial trapping and marking event in August. This observation suggests that dominant lobsters, with movement behavior characterized by smaller activity space, are more prone to locating a baited trap placed within their home range—and more prone to terminal entrapment once having located the baited trap (see above paragraph), as compared to their farther ranging and more submissive male conspecifics. Conversely, time spent in close proximity to a baited trap—as would be expected in cases where traps were deployed within the more limited home ranges of low home‐range individuals—seems to have translated into greater risk of capture. Similar observations were made by Monk and Arlinghaus (2017) in a recreational fin‐fish fishery. In their study, time spent in close proximity to fishing gear did relate to capture probability, while absolute encounter rate between individual fish and gear did not.

Our analyses demonstrated repeatability of behavioral traits (i.e., lobster personalities) suggesting that the movement metrics quantified from acoustic telemetry are indeed useful in capturing individual behavioral patterns. Moreover, it has implications for our interpretation of harvest selection acting on behavior and other correlated traits. A recent meta‐analysis found that approximately 50% of animal personality variation was attributable to additive genetic variation (Dochtermann et al., 2015). Thus, personality differences likely reflect genetic differences, and personality traits may evolve in response to selection. The existence of personality in a crustacean decapod is not an altogether novel finding. Since Briffa, Rundle, and Fryer (2008) reported behavioral consistency in the hermit crab *Pagurus bernhardus*, in both field and laboratory settings, a handful of studies have found evidence for personality in a range of crustacean species, including behaviors scored along a shy–bold axis (for a review, see Gherardi et al., 2012). Nonetheless, to the best of our knowledge, our study is unique in inferring repeatability of behavioral traits from long‐term, free‐ranging movement in a large‐bodied and commercially important decapod crustacean.

Natural selection has favored longevity and large body size in European lobster (Sheehy et al., 1999). Although some lobsters still do attain large size and old age under the present management regime, this potential is suppressed by the regulation imposing a minimum legal size (25 cm TL), above which a large proportion of the male population is removed each season. The extremely high annual fishing mortality exerted on catchable male lobsters tagged over the course of this study (2011:83%, 2012:76%) suggests that only a minor proportion of the catchable population will reach such a large and safe body size. From a Darwinian perspective on fisheries management (Dunlop, Enberg, Jørgensen, & Heino, 2009), it would make sense to allow more lobsters to realize their potentials with regard to growth and longevity, irrespective of behavioral type. One way of protecting against harvest selection is through introduction of lobster reserves or partially protected areas (PPAs) that ban capture of the species through gear restrictions. A network of marine protected areas established along the Norwegian Skagerrak coast in 2006 has demonstrated the usefulness of this management tool in rebuilding local lobster populations (Moland, Olsen, et al., 2013). In particular, lobster populations along the Norwegian Skagerrak coast have rebounded in relatively small PPAs, and the demography has shifted toward more large and old lobsters experiencing lowered levels of natural mortality (Moland, Olsen, et al., 2013; Moland, Ulmestrand, Olsen, & Stenseth, 2013). Beyond spatial protection, partial relaxation of the effect of harvest selection on body size and correlated traits can be obtained by the introduction of a maximum size limit (slot limit) in the fishery, and this approach was implemented in this system in 2017 (Sørdalen et al., 2018). How spatial protection via protected areas and slot limits combine to influence overall selection experienced by harvested species has yet to be explored but is a ripe area for understanding the selective and evolutionary implications of fishery management actions in this system.

In conclusion, our study shows how wild lobsters exposed to intense fishing are subject to a complex pattern of selection and highlight the need for field‐based, long‐term, and integrative studies of individual‐based characteristics as a fundamental step to ascertain how management actions exert selection in harvested populations.

## CONFLICT OF INTEREST

None declared.

## AUTHOR CONTRIBUTIONS

EM and EMO conceived the project; EM, JRW, and EMO collected the data; EM, EMO, SMC, JRW, and DVR analyzed the data; EM led the writing of the manuscript, and all authors contributed substantially and critically to the drafts and gave final approval for publication.

## DATA AVAILABILITY

Data for this study are available at the Dryad Digital Repository. https://doi.org/10.5061/dryad.5mr5k99 Data files: Lobster harvest selection.
