# Supplementary material for: Harvest selection on multiple traits in the wild revealed by aquatic animal telemetry
Source: Ecol Evol. 2019 May 20;9(11):6480–91. doi: 10.1002/ece3.5224 (PMC6580266; doi:10.1002/ece3.5224)
Supplement: Supplementary file 1 [file ECE3-9-6480-s001.docx]

**Supporting Information**

**Harvest selection on multiple traits in the wild revealed by aquatic animal telemetry**

Even Moland, Stephanie M. Carlson, David Villegas-Ríos, Jørgen Ree Wiig, Esben Moland Olsen

**Table SI1.** Summary of the Bayesian best fitted mixed-effect models explaining the variation in home range, cumulative distance, vertical position and depth amplitude. We provide summary statistics of the posterior distribution of each effect and MCMC p-values.

| **Response variable** | **Explanatory variable** | **Posterior mean** | **Lower 95% CI** | **Upper 95% CI** | **pMCMC** |
| --- | --- | --- | --- | --- | --- |
| Home range | Intercept | 11.955 | 11.691 | 12.229 | <0.001 |
|  | Month 10 | -0.166 | -0.357 | 0.031 | 0.091 |
|  | Month 11 | -0.413 | -0.665 | -0.156 | <0.001 |
| Cumulative distance | Intercept | 11.218 | 10.987 | 11.438 | <0.001 |
|  | Claw-width | 0.204 | 0.005 | 0.404 | 0.044 |
|  | Month 10 | -0.201 | -0.409 | 0.014 | 0.061 |
|  | Month 11 | -0.449 | -0.712 | -0.167 | 0.004 |
| Vertical position | Intercept | -564.689 | -1001.102 | -147.561 | 0.023 |
|  | Month 10 | 0.162 | -0.055 | 0.377 | 0.151 |
|  | Month 11 | 0.623 | 0.337 | 0.901 | <0.001 |
|  | Longitude | 0.001 | 0.0003 | 0.002 | 0.022 |
| Depth amplitude | Intercept | 2.205 | 1.910 | 2.530 | <0.001 |
|  | Month 10 | -0.172 | -0.372 | 0.004 | 0.072 |
|  | Month 11 | -0.729 | -0.988 | -0.472 | <0.001 |
|  | Claw-width | 0.764 | 0.197 | 1.325 | 0.011 |
|  | Year 2012 | -0.042 | -0.435 | 0.340 | 0.801 |
|  | Claw-width:Year 2012 | -0.758 | -1.398 | -0.198 | 0.013 |
